# Seed Germination Ecology of Feather Lovegrass [*Eragrostis tenella* (L.) Beauv. Ex Roemer & J.A. Schultes]

**DOI:** 10.1371/journal.pone.0079398

**Published:** 2013-11-08

**Authors:** Bhagirath S. Chauhan

**Affiliations:** Weed Scientist, Crop and Environmental Sciences Division, International Rice Research Institute, Los Baños, Metro Manila, Philippines; International Rice Research Institute, Philippines

## Abstract

Feather lovegrass [*Eragrostis tenella* (L.) Beauv. Ex Roemer & J.A. Schultes] is a C_4_ grass weed that has the ability to grow in both lowland and upland conditions. Experiments were conducted in the laboratory and screenhouse to evaluate the effect of environmental factors on germination, emergence, and growth of this weed species. Germination in the light/dark regime was higher at alternating day/night temperatures of 30/20 °C (98%) than at 35/25 °C (83%) or 25/15 °C (62%). Germination was completely inhibited by darkness. The osmotic potential and sodium chloride concentrations required for 50% inhibition of maximum germination were -0.7 MPa and 76 mM, respectively. The highest seedling emergence (69%) was observed from the seeds sown on the soil surface and no seedlings emerged from seeds buried at depths of 0.5 cm or more. The use of residue as mulches significantly reduced the emergence and biomass of feather lovegrass seedlings. A residue amount of 0.5 t ha^-1^ was needed to suppress 50% of the maximum seedlings. Because germination was strongly stimulated by light and seedling emergence was the highest for the seeds sown on the soil surface, feather lovegrass is likely to become a problematic weed in zero-till systems. The knowledge gained from this study could help in developing effective and sustainable weed management strategies.

## Introduction

Rice is an important crop in Asia, particularly in the Philippines, where it is mainly grown by transplanting of seedlings in ponded conditions. Because of labor and water scarcities, however, this method of rice establishment is being replaced by direct seeding of rice [1,2]. The spread of direct seeding and the repeated use of herbicides with a similar mode of action are shifting weed species populations in this rice ecosystem [3]. *Eragrostis* species are examples of such weed species that are increasing in direct-seeded rice systems and there are reports that these species are less affected by bispyribac-sodium, a common herbicide used as postemergence in Asia [4,5]. Bispyribac is an acetolactate synthase inhibitor, which can reduce the transport of photosynthate from source leaves to roots.

Feather lovegrass is one of the *Eragrostis* species that was reported to occur in dry-seeded and transplanting rice cultures in India and Thailand [6]. It is a common weed in upland rice in India, Indonesia, the Philippines, Thailand, and Vietnam [7]. In the Philippines, it has been reported as a common weed in both upland and lowland conditions [6]. Feather lovegrass is a C_4_ grass species, which occurs not only in crops but also in waste places, old walls, lawns, roadsides, beach dikes, and gardens [7]. It is a prolific seed producer and one plant can produce up to 140,000 seeds [8]. In addition, the weed is an alternate host for nematodes, viruses, and insects [7].

Despite the importance of this weed in different rice ecosystems, very little is known of its seed biology. The development of effective and sustainable weed management strategies depends on a detailed knowledge of weed seed biology [9,10]. Seed germination and seedling emergence of a weed species may be influenced by environmental factors, such as temperature, light, soil salinity, soil moisture, soil burial depth, and amount of crop residue present in the field. Light, for example, is one of the most significant ecological determinates for germination [11]. Seeds of weed species that require light for germination will germinate only when present on or near the soil surface. Such information and knowledge on seedling emergence at various burial depths could help in deciding on tillage systems to reduce emerging weed seedlings. Similarly, the use of crop residue in conservation agriculture systems may suppress the emergence of some weed species [12-14]. The response of weed seedling emergence to crop residue amounts may help to integrate different weed management components. In many Asian countries, rice is commonly grown in salt-affected and drought-prone areas, and the weed flora in these areas is often different. Information on the effect of salt and water stress on the germination of feather lovegrass could help predict the invasion potential of this species in such areas. A computer search of the available literature revealed no such information on lovegrass. 

A study was designed to determine the effects of temperature and light, salt and water stress, seed burial depth, and rice residue on the germination and emergence of feather lovegrass.

## Materials and Methods

### Seed collection and germination test

Seeds of feather lovegrass were collected in December 2012 from rice fields at the International Rice Research Institute, Los Baños, Laguna, Philippines. As the author works in this institute, no permission was needed to collect weed seeds. I also confirm that the field studies did not involve endangered or protected species. Seeds were collected from at least 200 plants, dried in a greenhouse for 2 days, bulked, cleaned, and stored in an airtight container at room temperature (25 °C) until used in the experiments (<3 months). Seed germination was determined after placing the seeds in 9-cm-diameter glass Petri dishes containing two layers of filter paper (Whatman No.1, Whatman plc, Springfield Mill, James Whatman Way, Maidstone, Kent ME14 twoLE, United Kingdom) and 5 ml of distilled water or treatment solution. Dishes were placed in an incubator at fluctuating day/night temperature of 30/20 °C in a light/dark regime, unless stated otherwise. The photoperiod was set at 12 hours to coincide with the higher temperature interval. Seed germination was assessed 15 days after the start of the experiment as no seeds germinated in a preliminary experiment in the growth chamber after this period. A seed was considered germinated when there was visible protrusion of the radicle.

### Effect of Temperature and Light on Germination

To determine the effect of temperature and light on germination, 25 seeds of feather lovegrass were placed in incubators set at three different day/night temperature regimes (25/15, 30/20, and 35/25 °C) in both light/dark and dark regimes. These temperature regimes were selected to reflect the temperature variation occurring in the Philippines. In the dark regime, the Petri dishes were wrapped in three layers of aluminum foil.

### Effect of water stress on germination

The effect of water stress on germination was assessed by incubating 25 seeds of feather lovegrass in Petri dishes with 5-ml solutions having osmotic potentials of -0.1, -0.2, -0.4, -0.6, -0.8, and -1.0 MPa. The solution concentrations were prepared by dissolving polyethylene glycol 8000 (Sigma-Aldrich Co., 3050 Spruce St., St. Louis, MO 63103) in distilled water [15].

### Effect of salt stress on germination

The effect of salinity on seed germination was determined by incubating 25 seeds of feather lovegrass in Petri dishes with 5-ml solutions of 0, 25, 50, 100, 150, 200, and 250 mM of sodium chloride (NaCl, Mallinckrodt Baker Inc., Phillipsburg, NJ).

### Effect of seed burial depth on seedling emergence

This experiment was conducted in a screenhouse with an overhead transparent plastic cover to avoid rain damage. Fifty seeds of feather lovegrass were placed on the soil surface or covered with the same soil to achieve burial depths of 0.5, 1, 2, and 3 cm in plastic trays (8 cm x 8 cm x 5 cm). The soil used in this experiment had clay, silt, and sand contents of 32, 37, and 31%, respectively. Before being placed in the trays, the soil was autoclaved and passed through a 3-mm sieve. The trays were watered initially with an overhead “mist” sprinkler and later subirrigated. Seedlings were considered emerged when a cotyledon could be visibly distinguished. The experiment was terminated 28 days after planting. 

### Effect of rice residue amount on seedling emergence and seedling biomass

This experiment was conducted in the screenhouse and the soil used was the same as described earlier for the seed burial depth experiment. Fifty seeds of feather lovegrass were sown on the soil surface in plastic pots (15-cm-diameter and 14.5-cm height). Air-dried rice residue (leaves and stems) of cultivar NSICRc222 was uniformly spread on the soil surface at rates equivalent to 0, 1, 2, 4, and 6 t ha^-1^. The depth of mulch at these residue amounts corresponded to approximate depths of 0.3, 0.6, 1.3, and 1.8 cm, respectively. Pots were watered initially with an overhead mist sprinkler and later subirrigated. The emerged seedlings above the mulch surface were counted 28 days after sowing. Emerged seedlings were harvested, placed in paper bags, and, oven-dried at 70 °C for 72 hours for measuring dry biomass.

### Statistical analyses

All the experiments were conducted in a randomized complete block design and each treatment was replicated four times. All experiments were repeated over time and the second run of experiments started within a month of termination of the first run. ANOVA was performed on nontransformed data because transformation did not improve the homogeneity of variance. The data from the two experimental runs were combined for analysis (a total of eight replications) because there were no differences between the two runs. The data on the burial depth and temperature and light experiments were subjected to ANOVA and means were separated using LSD at *P* = 0.05 (GenStat 13th Edition, VSN International Ltd., United Kingdom). For other experiments, regression analysis was done using SigmaPlot 10.0 (Systat Software, Inc., Point Richmond, CA). The means of each data point were shown with standard error of mean (standard deviation of mean divided by the number of replications) with regression analysis.

Values for germination (%) at different concentrations of osmotic potential were best fitted to the linear model 

y=ax+b

where *y* represents germination (%) at osmotic potential *x*. A three-parameter sigmoid model 

y=Gmax/{1+exp[−(x−C0)/b]}

was fitted to the seed germination (%) obtained at different salt concentrations, where *y* is the total germination (%) at NaCl concentration *x*, *Gmax* is the maximum germination (%), *C0* is the NaCl concentration required for 50% inhibition of maximum germination, and *b* indicates the slope. An exponential curve 

y=R.exp(−Rrate.x)

was fitted to the seedling emergence (%) or biomass (g) obtained at different residue amounts, where *y* represents seedling emergence (%) or seedling biomass (g) at residue amount *x*, *R* is the maximum emergence or biomass, and *Rrate* is the slope.

## Results and Discussion

### Effect of temperature and light on germination

**Figure 1:**
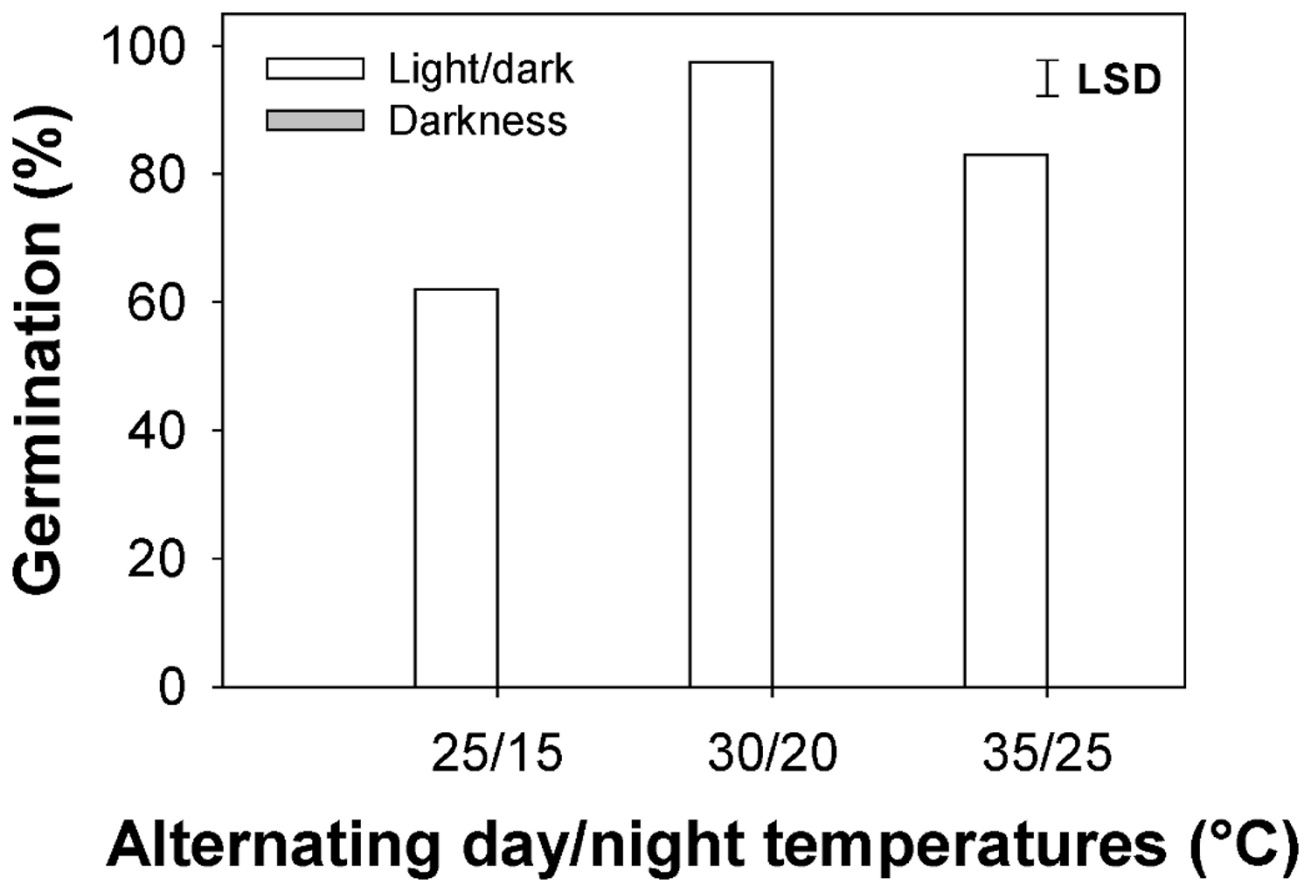
Effect of alternating day/night temperature and light on germination of feather lovegrass seeds after 15 days of incubation. No germination occurred in the dark regime.

There was an interaction of temperature and light in the germination of feather lovegrass (Figure 1). Light strongly stimulated germination and dark completely inhibited germination of this weed species. When exposed to alternating temperatures in the light/dark regime, feather lovegrass germinated at all the tested temperature ranges. Maximum germination (97.5%) occurred when seeds were exposed to the 30/20 °C regime and the germination in this temperature regime was higher than the germination at 35/25 °C (83%) and 25/15 °C (62 %). Germination in the 25/15 °C temperature regime was significantly lower than at higher temperatures.

Temperature is considered an important factor influencing seed germination of several weed species. Some weed species can germinate over a wide temperature range [16,17]. Feather lovegrass germinated at all the tested ranges of temperature; however, germination was lower in suboptimal conditions, that is, at 25/15 °C fluctuating day/night temperature. The lower germination of feather lovegrass at 25/15 °C temperature in our study suggests that this weed might be a less problematic weed in the cooler portion of the growing season or at high altitudes in tropical areas.

Light plays a significant role in seed germination of many weed species. The response of germination to light is often species-specific [10]. Seeds of some weed species [e.g., tropical signalgrass (*Urochloa subquadripara* (Trin.) R.D. Webster)] germinate equally in light and dark conditions [18] and seeds of other species [e.g., Chinese sprangletop (*Leptochloa chinensis* (L.) Nees) and barnyardgrass (*Echinochloa crus-galli* (L.) Beauv.)] require light to stimulate germination [3,19]. In our study, feather lovegrass had an absolute light requirement for germination. Such a result demonstrates that seeds of this weed species are positively photoblastic. The ecological significance of such a response to light is that light acts as a soil depth indicator, suggesting higher germination for seeds present on or near the soil surface than seeds buried deep in the soil [20]. An earlier study, in fact, suggested that weed species that require light to stimulate germination have the potential to become problematic weeds in no-till systems as most of the weed seeds remain on the soil surface after crop planting in these systems [21]. On the other hand, the built-up seed bank of such weed species in no-till systems could be depleted by the use of stale seedbed practices before crop planting [1,10].

### Effect of Water Stress on Germination

**Figure 2:**
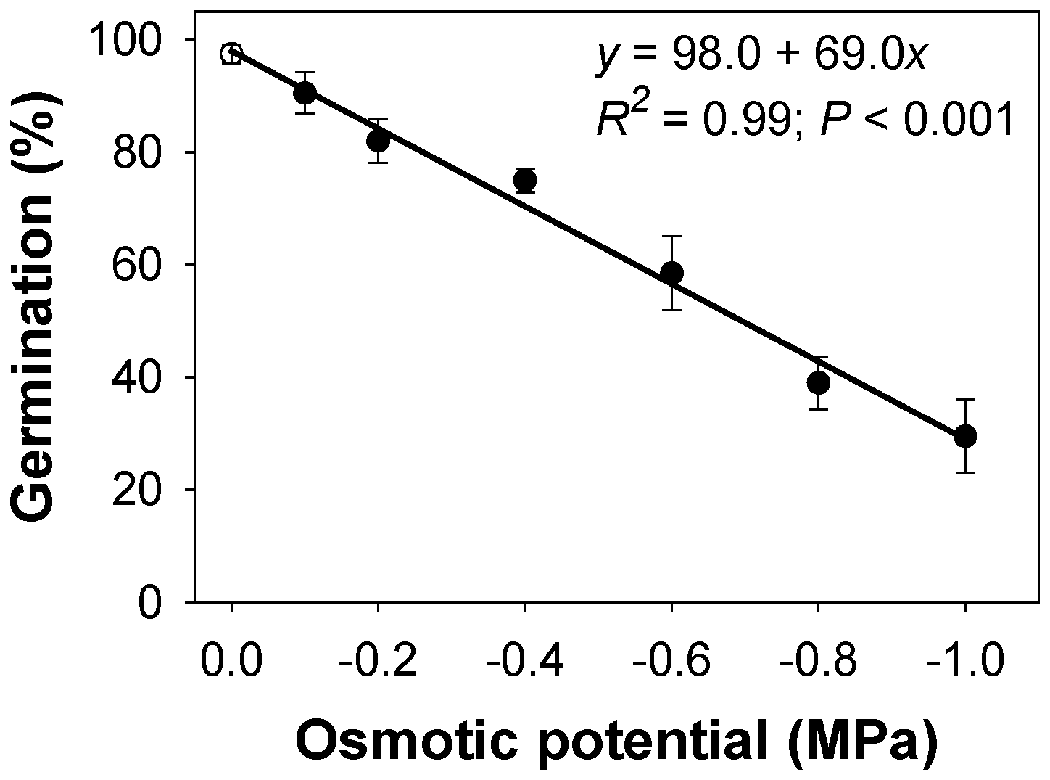
Effect of osmotic potential on germination of feather lovegrass seeds after 15 days of incubation at 30/20 °C day/night temperature. The line represents a linear model, *y*=(*ax*+*b*), fitted to the data, where *y* represents germination (%) at osmotic potential *x*.

The germination percentage of feather lovegrass as a function of a range of osmotic potentials is shown in Figure 2. Germination of seeds exposed to no stress (0 MPa) was 98%; however, seed germination declined linearly with increases in osmotic potential concentrations. Some seeds germinated even at -1.0 MPa, suggesting that feather lovegrass can even germinate under moderately water-stressed conditions that would make feather lovegrass competitive in a crop that is susceptible to drought stress, such as rice. The osmotic potential required for 50% inhibition of maximum germination of feather lovegrass was -0.7 MPa. In itchgrass [*Rottboellia cochinchinensis* (Lour.) W.D. Clayton] and southern crabgrass [*Digitaria ciliaris* (Retz.) Koel], the osmotic potentials required for 50% inhibition of their maximum germination were -0.6 and -0.4 MPa, respectively [22,23]. 

### Effect of salt stress on germination

**Figure 3:**
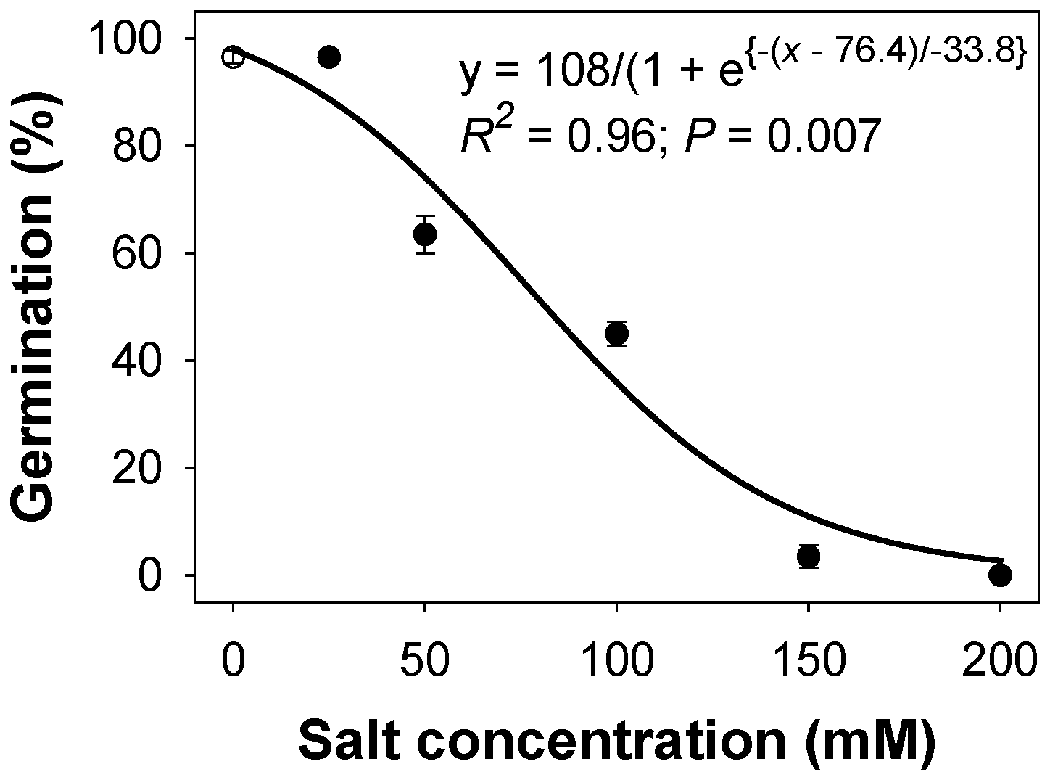
Effect of salt (sodium chloride, NaCl) concentration on germination of feather lovegrass seeds after 15 days of incubation at 30/20 °C day/night temperature. The line represents a three-parameter sigmoid model, *y*=*Gmax*/{1+*exp*[−(*x*−*C*0)/*b*]}, fitted to the data, where *y* is the total germination (%) at NaCl concentration *x*, *Gmax* is the maximum germination (%), *C0* is the NaCl concentration required for 50% inhibition of maximum germination, and *b* indicates the slope.

A sigmoid response was observed in the germination of feather lovegrass seeds with increases in NaCl concentration from 0 to 200 mM (Figure 3). Maximum germination was achieved when seeds were incubated in no-stress conditions. Germination was completely inhibited at 200 mM NaCl. The concentration required for 50% inhibition of maximum germination was 76 mM NaCl. Similar results were reported for goosegrass [*Eleusine indica* (L.) Gaertn.], in which 78 mM NaCl inhibited 50% of maximum germination [24]. Salt stress is an important abiotic constraint in cereal production worldwide, including Asia [25]. The data of our study suggest that feather lovegrass could germinate at high soil salinity and this could be an important characteristic that would allow this weed species to colonize saline areas.

### Effect of seed burial depth on seedling emergence

Seedling emergence of feather lovegrass was significantly affected by seed burial depth. Seedling emergence was the highest (69% ± 2%) for seeds placed on the soil surface, and no seedlings emerged at burial depths of 0.5 cm or more.

There are several possible explanations for the lack of emergence from seeds buried at deeper depths; however, limited light penetration in the soil is probably the main reason for no emergence from buried weed seeds. Seeds buried below 2 mm usually receive a very limited proportion of incident light, which is not enough to trigger germination [26]. The response of seedling emergence to burial depth is also consistent with the response of germination to light, as there was an absolute requirement of light for the germination of feather lovegrass seeds. Small seed size, hypoxia, and low rates of gaseous diffusion at deeper depths could also be responsible for the lack of emergence [27,28]. Small-seeded species, such as feather lovegrass (27 mg per 1000 seeds), might have insufficient seed reserves to support seedling emergence from deeper depths. Similar results were reported for Chinese sprangletop, in which no seedlings emerged from a burial depth of 0.5 cm or more [3]. The absolute requirement of light for germination was suggested as one of the reasons for such a response to burial depths.

The results of this experiment suggest that, under field conditions, no-till farming practices or practices that achieve shallow burial of weed seeds would increase the emergence of feather lovegrass. In no-till or conservation agriculture practices, most of the weed seeds remain on or near the soil surface after crop planting [29]. The weed seeds present on the soil surface, however, are prone to predation, mortality, and stale seedbed practices. The buildup of a weed seed bank on the soil surface can also be managed by a deep tillage operation that buries weed seeds below their maximum depth of emergence.

### Effect of rice residue amount on seedling emergence and seedling biomass

**Figure 4:**
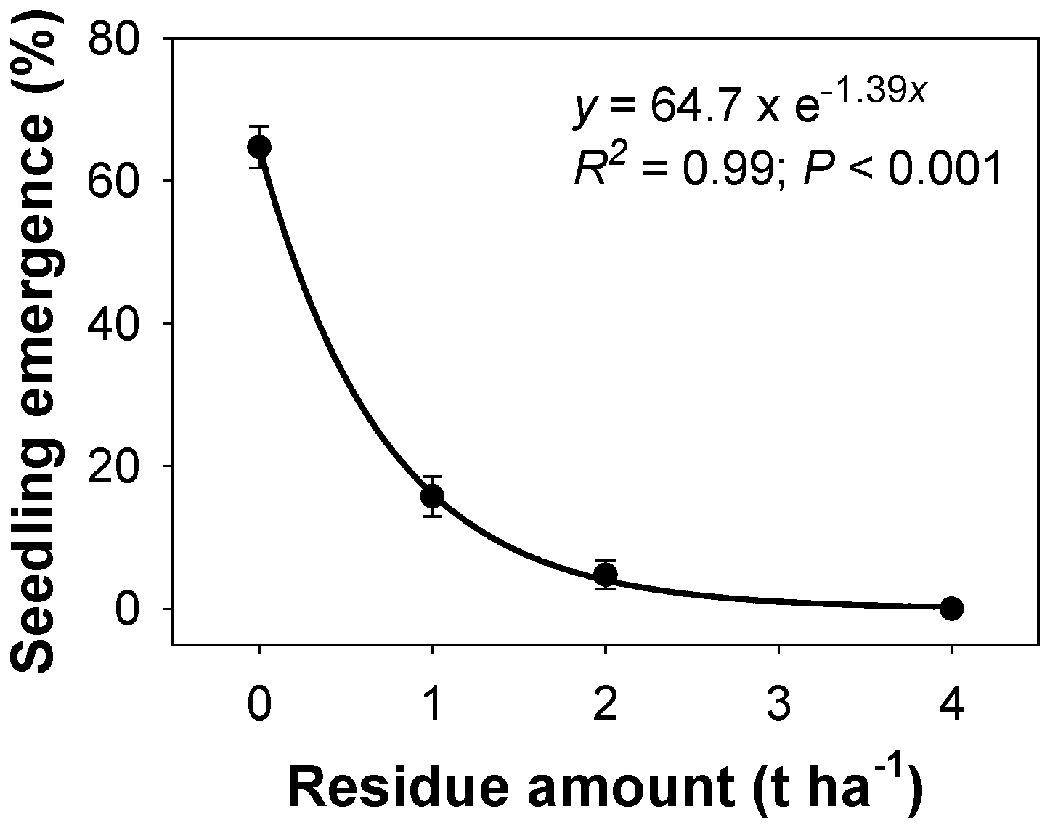
Effect of rice residue amount on seedling emergence of feather lovegrass at 28 days after sowing. The line represents an exponential model, *y*=*R*.*exp*(−*Rrate*.*x*), fitted to the data, where *y* represents seedling emergence (%) at residue amount *x*, *R* is maximum emergence, and *Rrate* indicates the slope.

**Figure 5:**
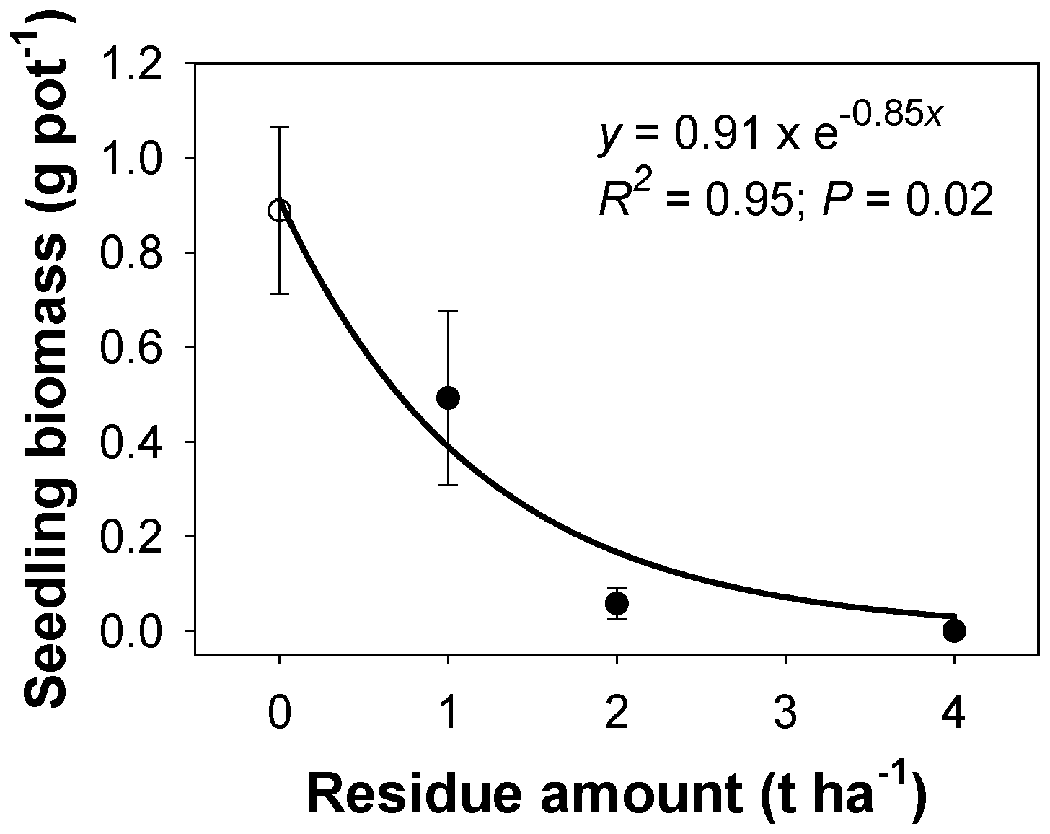
Effect of rice residue amount on seedling biomass of feather lovegrass at 28 days after sowing. The line represents an exponential model, *y*=*R*.*exp*(−*Rrate*.*x*), fitted to the data, where *y* represents seedling biomass (g) at residue amount *x*, *R* is maximum biomass, and *Rrate* indicates the slope.

Seedling emergence and seedling biomass of feather lovegrass declined exponentially with the addition of residue (Figures 4 and 5). Seedling emergence was highest (65%) in the absence of residue, and this declined sharply with increases in the rice residue amount (Figure 4). An amount of 0.5 t ha^-1^ of residue was needed to suppress 50% of maximum emergence. No seedlings emerged with the addition of 4 t ha^-1^ or more residue. Similar to the response of seedling emergence, maximum seedling biomass (0.9 g pot^-1^) was obtained in the absence of residue, and biomass declined sharply with the increasing residue amounts (Figure 5). The amount of residue to suppress 50% of maximum biomass was 0.8 t ha^-1^.

The use of crop residue as mulches can suppress seedling emergence and growth of several weed species [10,14,30,31]. A recent field study in the Philippines reported suppression of the emergence and growth of *Eclipta* [*Eclipta prostrata* (L.) L.], crowfootgrass [*Dactyloctenium aegyptium* (L.) Willd.], junglerice [*Echinochloa colona* (L.) Link], and Chinese sprangletop with the use of 6 t ha^-1^ of rice residue in a sprinkler-irrigated zero-till dry-seeded rice system [13]. Rice cultivars may have allelopathic potential [32]; however, the cultivar used in our experiment is not known for its allelopathic effects. Therefore, reductions in light transmittance and temperature fluctuations, and residue cover being a physical barrier, could have been responsible for the reduced emergence and growth with the addition of rice residue [33,34].

The results suggest that the use of residue as mulches can be integrated with other weed management strategies to achieve effective weed control. This approach would be very helpful for farmers who practice organic agriculture or who do not have enough resources to buy herbicides [13]. The use of residue in the field will also help in reducing environmental pollution caused by frequent burning of rice residue in the field, for example, in India and the Philippines [1,35]. In Asia, seeding machines are already available that can plant seeds into anchored and loose residue of up to 7-8 t ha^-1^ [35].

In summary, the germination of feather lovegrass was strongly stimulated by light. The highest seedling emergence was observed from seeds placed on the soil surface and seedlings could not emerge from burial depths of 0.5 cm or more. The use of rice residue as mulch suppressed seedling emergence and biomass of feather lovegrass. Seed germination was moderately tolerant of salt and water stress conditions.

## References

[1] Chauhan BS (2012) Weed ecology and weed management strategies for dry-seeded rice in Asia. Weed Technol 26: 1-13. doi:10.1614/WT-D-11-00105.1.

[2] Pandey S, Velasco L (2005) Trends in crop establishment methods in Asia and research issues, in Toriyama K Heong KL Hardy B, Rice Is Life: Scientific Perspectives for the 21st Century. Los Baños, Philippines: International Rice Research Institute and Tsukuba, Japan: Japan International Research Center for Agricultural Sciences, p. 178-181.

[3] Chauhan BS, Johnson DE (2008) Germination ecology of Chinese sprangletop (*Leptochloa* *chinensis*) in the Philippines. Weed Sci 56: 820-825. doi:10.1614/WS-08-070.1.

[4] Gopal R, Jat RK, Malik RK, Kumar V, Alam MM et al. (2010) Direct dry seeded rice production technology and weed management in rice based systems. Technical Bulletin. New Delhi, India: International Maize and Wheat Improvement Center. 28 pp.

[5] Kumar V, Ladha JK (2011) Direct seeding of rice: recent developments and future research needs. Adv Agron 111: 299-413.

[6] Moody K (1989) Weeds Reported in Rice in South and Southeast Asia. Los Baños, Laguna, Philippines: International Rice Research Institute. 442 pp.

[7] Galinato MI, Moody K, Piggin CM (1999) Upland rice weeds of South and Southeast Asia., Hardy B Makati City, (Philippines): International Rice Research Institute p. 156.

[8] Pancho JV (1964) Seed sizes and production capacities in common weed species of the rice fields of the Philippines. Philipp Agric 48: 307-316.

[9] Bhowmik PC (1997) Weed biology: importance to weed management. Weed Sci 45: 349-356.

[10] Chauhan BS, Johnson DE (2010) The role of seed ecology in improving weed management strategies in the tropics. Adv Agron 105: 221-262. doi:10.1016/S0065-2113(10)05006-6.

[11] Crisraudo A, Gresta F, Luciani F, Resticcia A (2007) Effects of after-harvest period and environmental factors on seed dormancy of *Amaranthus* species. Weed Res 47: 327-334. doi:10.1111/j.1365-3180.2007.00574.x.

[12] Buhler DD, Mester TC, Kohler KA (1996) The effect of maize residues and tillage on emergence of *Setaria* *faberi*, *Abutilon* *theophrasti*, *Amaranthus* *retroflexus* and *Chenopodium* *album* . Weed Res 36: 153-165. doi:10.1111/j.1365-3180.1996.tb01811.x.

[13] Chauhan BS, Abugho SB (2013) Effect of crop residue on seedling emergence and growth of selected weed species in a sprinkler-irrigated zero-till dry-seeded rice system. Weed Sci 61: 403-409. doi:10.1614/WS-D-12-00147.1.

[14] Teasdale JR, Beste CE, Potts WE (1991) Response of weeds to tillage and cover crop residue. Weed Sci 39: 195-199.

[15] Michel BE (1983) Evaluation of the water potentials of solutions of polyethylene glycol 8000 both in the absence and presence of other solutes. Plant Physiol 72: 66-70. doi:10.1104/pp.72.1.66. PubMed: 16662983. 16662983 PMC1066170

[16] Benvenuti S, Dinelli G, Bonetti A (2004) Germination ecology of *Leptochloa* *chinensis*: a new weed in the Italian agro-environment. Weed Res 44: 87-96. doi:10.1111/j.1365-3180.2003.00376.x.

[17] Burke IC, Thomas WE, Spears JF, Wilcut JW (2003) Influence of environmental factors on after-ripened crowfootgrass (*Dactyloctenium* *aegyptium*) seed germination. Weed Sci 51: 342-347. doi:10.1614/0043-1745(2003)051[0342:IOEFOA]2.0.CO;2.

[18] Teuton TC, Brecke BJ, Unruh JB, MacDonald GE, Miller GL et al. (2004) Factors affecting seed germination of tropical signalgrass (*Urochloa* *subquadripara*). Weed Sci 52: 376-381. doi:10.1614/WS-03-121R1.

[19] Boyd NS, Van Acker RC (2004) Seed germination of common weed species as affected by oxygen concentration, light, and osmotic potential. Weed Sci 52: 589-596. doi:10.1614/WS-03-15R2.

[20] Schutz W, Milberg P, Lamont BB (2002) Seed dormancy, after-ripening and light requirements of four annual Asteraceae in south-western Australia. Ann Bot 90: 707-714. doi:10.1093/aob/mcf250. PubMed: 12451026. 12451026 PMC4240361

[21] Cousens RD, Baweja R, Vaths J, Schofield M (1993) Comparative biology of cruciferous weeds: a preliminary study. In Proceedings of the 10th Australian and 14th Asian-Pacific Weed Conference: Brisbane, Australia: Weed Society of Queensland, editor. pp. 376-380

[22] Bolfrey-Arku GE-K, Chauhan BS, Johnson DE (2011) Seed germination ecology of itchgrass (*Rottboellia* *cochinchinensis*). Weed Sci 59: 182-187. doi:10.1614/WS-D-10-00095.1.

[23] Chauhan BS, Johnson DE (2008) Germination ecology of southern crabgrass (*Digitaria* *ciliaris*) and India crabgrass (*Digitaria* *longiflora*): two important weeds of rice in tropics. Weed Sci 56: 722-728. doi:10.1614/WS-08-049.1.

[24] Chauhan BS, Johnson DE (2008) Germination ecology of goosegrass (*Eleusine* *indica*): an important grass weed of rainfed rice. Weed Sci 56: 699-706. doi:10.1614/WS-08-048.1.

[25] Lafitte HR, Ismail A, Bennett J (2006) Abiotic stress tolerance in tropical rice: progress and future prospects. Oryza 43: 171-186.

[26] Woolley JT, Stoller EW (1978) Light penetration and light-induced seed germination in soil. Plant Physiol 61: 597-600. doi:10.1104/pp.61.4.597. PubMed: 16660344. 16660344 PMC1091925

[27] Baskin CC, Baskin JM (1998) Seeds: Ecology, Biogeography, and Evolution of Dormancy and Germination. San Diego, CA: Academic p. 666.

[28] Benvenuti S (2003) Soil texture involvement in germination and emergence of buried weed seeds. Agron J 95: 191-198. doi:10.2134/agronj2003.0191.

[29] Chauhan BS, Singh RG, Mahajan G (2012) Ecology and management of weeds under conservation agriculture: a review. Crop Protect 38: 57-65. doi:10.1016/j.cropro.2012.03.010.

[30] Crutchfield DA, Wicks GA, Burnside OC (1985) Effect of winter wheat (*Triticum* *aestivum*) straw mulch level on weed control. Weed Sci 34: 110-114.

[31] Mohler CL (1991) Effects of tillage and mulch on weed biomass and sweet corn yield. Weed Technol 5: 545-552.

[32] Mahajan G, Chauhan BS (2013) The role of cultivars in managing weeds in dry-seeded rice production systems. Crop Protect 49: 52-57. doi:10.1016/j.cropro.2013.03.008.

[33] Mohler CL, Calloway MB (1992) Effects of tillage and mulch on the emergence and survival of weeds in sweet corn. J Appl Ecol 29: 21-34. doi:10.2307/2404343.

[34] Teasdale JR, Mohler CL (1993) Light transmittance, soil temperature, and soil moisture under residue of hairy vetch and rye. Agron J 85: 673-680. doi:10.2134/agronj1993.00021962008500030029x.

[35] Gupta RK, Ladha JK, Singh S, Singh R, Jat ML et al. (2006) Production technology for direct seeded rice. In Rice-Wheat Consortium, Technical Bulletin 8. Rice-Wheat Consortium for the Indo-Gangetic Plains: New Delhi, India 16 p..

